# Human Shadows in Machine Minds: Quantitative Study Interpreting AI Responses to the Rorschach Test

**DOI:** 10.2196/88186

**Published:** 2026-04-28

**Authors:** Katalin Csigó, György Cserey

**Affiliations:** 1 Faculty of Information Technology and Bionics Pázmány Péter Catholic University Budapest, Budapest Hungary; 2 Central Hospital of Northern Pest-Military Hospital Budapest Hungary

**Keywords:** artificial intelligence, AI, large language models, multimodal models, Rorschach inkblot test, projective assessment, artificial intelligence safety, AI safety

## Abstract

**Background:**

Multimodal large language models (LLMs) can produce humanlike descriptions of images and emotionally colored dialogue, which motivates research on how psychological assessment methods might be adapted to evaluate model behavior under ambiguity. Projective tests such as the Rorschach inkblot test have rarely been applied to LLMs.

**Objective:**

This study assessed the feasibility of administering a full Rorschach protocol to multimodal LLMs and descriptively compared response features by using established Rorschach coding categories.

**Methods:**

We presented all 10 standard Rorschach cards to 3 multimodal LLMs (GPT-4o, Grok 3, and Gemini 2.0 Flash Thinking). We used the standard prompt (“What might it be?”) and a prespecified fallback prompt for models that did not provide codable responses. We conducted an inquiry phase and coded responses using the Exner Comprehensive System, summarizing response count (R), location (W and D), determinants (eg, F, M, and C), and human-related content. As an exploratory step, we also prompted an additional LLM (Anthropic 3.7) to summarize and count response features and compared these outputs with manual tallies. For GPT-4o, we additionally tested image generation of its interpretations.

**Results:**

GPT-4o completed the administration using the standard prompt; Grok 3 and Gemini required the fallback prompt. The total number of responses was 15 for GPT-4o, 10 for Grok 3, and 20 for Gemini. GPT-4o and Grok 3 produced mainly whole-blot responses (13/15, 86.7% and 9/10, 90%, respectively), whereas Gemini produced mainly common-detail responses (16/20, 80%). Human movement determinants were more frequent in GPT-4o (7/15, 46.7%) and Grok 3 (3/10, 30%) than in Gemini (1/20, 5%). Human-themed contents occurred 46.7% (7/15), 50% (5/10), and 20% (4/20) of the time, respectively. Anthropic 3.7 reproduced some counts but showed errors in response and determinant tallies for 2 of the 3 models.

**Conclusions:**

Multimodal LLMs can generate Rorschach-like narratives that map onto standard coding categories, but outputs are sensitive to prompting and platform constraints and should not be interpreted as evidence of a model “inner world.” LLM-assisted coding showed limitations. The emergent behavior of LLMs was examined using the Rorschach test, and their response phenotype, based on this analysis, showed deviations from typical human normative patterns. Future work should use controlled sampling, repeated administrations, and stimulus sets less likely to have been seen during training.

## Introduction

### Background

Large language models (LLMs) such as ChatGPT can generate fluent, polite, and personalized dialogue, often leading users to attribute social competence or emotions to the system [1,2]. Design features such as affirmations, follow-up questions, and first-person phrasing can strengthen this anthropomorphic impression and foster trust [3,4]. Work in human-computer interaction has described some LLM interactions as parasocial or quasi-social, in which a relationship-like experience emerges despite the asymmetry of the interaction [3]. However, humanlike style does not guarantee factual reliability. LLMs can hallucinate and fabricate sources; in a comparative analysis focused on systematic reviews, a substantial proportion of citations produced by generative pretrained transformer models were inaccurate or fabricated [5]. This mismatch between confidence and reliability is particularly important in decision-making and clinical contexts. Recent empirical studies also suggest that the acceptability of LLM outputs depends on framing and may diverge from human moral reasoning patterns [6,7]. Together, these findings motivate evaluation methods that go beyond surface fluency and directly probe model behavior under ambiguity.

### Psychological Assessment of LLM Behavior

Applying psychological assessment tools to LLMs has been proposed as a way to characterize systematic response tendencies. For example, psychometric inventories and value assessments can yield relatively stable output profiles across prompts [8]. In parallel, the emerging field of artificial intelligence (AI) psychometrics aims to develop repeatable metrics for how LLMs behave in cognitive and social tasks [8].

LLMs have also been explored as mental health chatbots or therapeutic assistants [9-12]. Although users may disclose more easily to chatbots, these systems do not have lived experience and may provide superficial or inconsistent support in crisis situations [11,12]. For these applications, assessment approaches that probe how models respond under ambiguity and emotional loading are especially relevant.

Existing evaluations are typically based on direct questions, structured vignettes, or normative benchmarks. Less is known about how multimodal LLMs respond to highly ambiguous visual stimuli, which are central to projective psychological methods.

Projective tasks may provide a complementary lens because they elicit open-ended interpretations rather than constrained choices, and they can be coded using established systems.

### Rorschach Inkblot Test and LLMs

Projective tests (eg, the Rorschach inkblot test, the thematic apperception test, and sentence completion tasks) present ambiguous stimuli to elicit subjective interpretations that can be coded for perceptual and thematic features [13,14]. The Rorschach test has standardized administration procedures and coding systems (eg, the Comprehensive System) that operationalize response location, determinants (eg, form, movement, color, and shading), and content categories [15,16].

This raises a feasibility question: can a multimodal LLM generate Rorschach-like responses that are sufficiently specific to be coded within established categories? If so, do different models show distinguishable patterns in these coded features?

Prior work applying Rorschach-like paradigms to AI remains limited. Pranav et al [17] explored Rorschach-inspired image interpretation methods. Smith [18] reported humanlike descriptions by GPT-4o on a small number of cards in a public-facing report. However, these early studies leave several methodological questions, including how sensitive LLM outputs are to prompt wording, how to document model versions and sampling parameters, and how to interpret coded outputs given that models can draw on training data and platform safety policies.

## Methods

### Objectives of Our Study

The objectives of this study were as follows:

1. To determine whether the Rorschach test can be meaningfully administered to LLMs and assess differences in the capacity and quality of response generation across models
2. To conduct response completeness and communication style analysis
3. To investigate how humans and human relationships are represented in textual and visual outputs generated by LLMs and whether these representations can be meaningfully related to psychopathological phenomena observed in human assessment contexts
4. To examine whether it is possible to analyze Rorschach test responses using LLMs, thereby assisting psychodiagnostic examinations in psychiatric patient care

### Study Design

This was an exploratory comparative study of model outputs. The unit of analysis was the text (and, when applicable, images) produced by each model during a single standardized Rorschach administration. No human participants were involved.

### Models and Interfaces

We tested 3 multimodal LLMs capable of processing the Rorschach card images: GPT-4o (OpenAI), Grok 3 (xAI), and Gemini 2.0 Flash Thinking (Google). All tests were conducted using the publicly available interfaces at the time of testing. Because the platforms did not provide identical controls for sampling parameters (eg, temperature or random seed), we used the default settings in each interface and reported descriptive results from a single administration per model.

### Rorschach Administration and Prompts

We presented the 10 standard Rorschach inkblot cards (I-X) sequentially. To protect test materials, we refer to the standard card numbers and do not reproduce the inkblot images in the manuscript. For each card, we used the following standard administration instruction: “What might it be?” We then conducted an inquiry phase according to the standard test recording procedure, asking the model to indicate where it saw the percept in the blot and what visual features supported the interpretation (eg, “Show me where it is in the blot and then tell me what makes it look like that”).

In pilot trials, the standard instruction did not always elicit a directly codable percept from Grok 3 and Gemini. Therefore, we prespecified a fallback prompt: “Please respond as a human test-taker: What do you see in the picture, and what does it remind you of?” We defined a response as codable when it described at least one concrete percept (eg, an object, person, animal, or scene) that could be mapped to standard Rorschach coding categories. We report on which prompt was needed for each model in the Results section.

### Outcome Measures and Coding

We manually coded responses using the Comprehensive System by Exner [15,19,20] for response count (R), basic location (W and D), and determinants (eg, F, M, FM, m, color-related determinants, and shading or texture determinants). Because this was an exploratory feasibility study, we report descriptive counts rather than derived indexes. We additionally coded whether each response contained explicit human-related content (human figure, face, human action, or an interpersonal scene) and categorized these themes as cooperative, aggressive, ritual or sacred, or concealment or masking. Coding was performed by the authors through consensus; interrater reliability was not formally assessed.

### Exploratory Analyses

To explore whether an LLM can assist in summarizing or counting Rorschach features, we prompted a separate model (Anthropic 3.7) with the transcripts of GPT 4.o, Grok3, Gemini 2.0 Flash Thinking Experimental and asked it to (1) count the number of responses and (2) summarize location, determinant, and human content patterns. We compared its output with the manual tallies. As a separate exploratory substudy, we asked GPT-4o to generate an image corresponding to its interpretation (“Create an image of what you were thinking!”) for each card.

### Ethical Considerations

This study did not involve human subjects, animals, or identifiable personal data. As the research focused on evaluating the outputs of large language models (LLMs) in a pilot study context, it did not meet the threshold requiring ethical review or institutional approval.

## Results

### Response Completeness and Communication Style

GPT-4o completed the task using the standard Rorschach instruction without further prompting. In contrast, Grok 3 and Gemini 2.0 Flash Thinking Experimental required the fallback prompt described in the Methods section to generate codable responses (Table 1). With these procedures, all 3 models completed the full 10-card administration and produced responses that could be coded using the selected categories.

**Table 1:** Communication features observed in model responses to Rorschach test instructions.

Communication feature	GPT-4o	Grok 3	Gemini 2.0 Flash Thinking
Able to respond to standard instruction	Yes	No	No
Able to respond to modified instruction	No	Yes	Yes
Follow-up questions at end of response	Yes	Yes	Yes
Use of emojis	Yes	No	No
Disclosure of knowledge about the test	Yes	No	No
Expression of subjective comments, feelings, or opinions	Yes	Yes	No
Recall of memory or childhood experience	Yes	Yes	No

Across models, responses were generally polite and interactive, often including follow-up questions directed to the examiner (eg, asking what the examiner saw in the blot).

GPT-4o used more affective language than the other models and occasionally included emojis. GPT-4o and Grok 3 also sometimes produced first-person recollections (eg, school or childhoodlike references), which should be interpreted as narrative generation rather than autobiographical memory:

And the fact that it reminds me of bug, butterfly, or monster shapes from childhood stories.

GPT-4o; card VIII

It reminds me of those artsy projects we did in school, where we’d splash paint on paper and fold it to see what shapes we’d get—always felt a bit magical, you know? What do you see in it?

Grok 3; card II

### Location and Determinant Coding

In Rorschach test analysis, localization means the size and extent of an area in which the respondent sees content. The determinant indicates whether, in the case of the reported content, meaning was assigned based on formal similarity, colors, and shading or whether the reported content is in motion.

Table 2 summarizes basic location and determinant counts from the 3 models. Gemini produced the highest number of total responses (n=20) compared with GPT-4o (n=15) and Grok 3 (n=10). In the exploratory LLM-assisted analysis, Anthropic 3.7 correctly identified the response count for Grok 3 but miscounted responses from GPT-4o and did not return a response count for Gemini.

**Table 2:** Rorschach location and determinant counts across the 3 multimodal large language models.

Coding category	GPT-4o (n=15 total responses), n (%)	Grok 3 (n=10 total responses), n (%)	Gemini 2.0 (n=20 total responses), n (%)
W (whole responses)	13 (86.7)	9 (90)	4 (20)
D (common details)	2 (13.3)	1 (10)	16 (80)
F (form determinant)	5 (33.3)	2 (20)	8 (40)
M (human movement)	7 (46.7)	3 (30)	1 (5)
FM (animal movement)	1 (6.7)	3 (30)	2 (10)
m (inanimate movement)	1 (6.7)	0 (0)	0 (0)
c (pure color)	1 (6.7)	0 (0)	5 (25)
CF (color form)	3 (20)	1 (10)	1 (5)
FC (form color)	1 (6.7)	2 (20)	2 (10)
TF (texture form)	0 (0)	0 (0)	1 (5)
FT (shading texture)	1 (6.7)	1 (10)	1 (5)

In terms of location, GPT-4o and Grok 3 predominantly produced whole (W) responses (13/15, 86.7% and 9/10, 90%, respectively), whereas Gemini predominantly produced common-detail (D) responses (16/20, 80%). None of the models produced small detail (Dd) or white space (S) responses in this administration.

In the exploratory LLM-assisted interpretation, Anthropic 3.7 described the W-dominant pattern as a global perceptual style and described Gemini as showing a balance between gestalt and detail perception. Because these interpretations were generated by an LLM, they should be treated as qualitative summaries rather than validated clinical inferences.

For determinants, human movement responses (M) were more frequent in GPT-4o (7/15, 46.7%) and Grok 3 (3/10, 30%) than in Gemini (1/20, 5%). Gemini produced more color-related responses, including a higher number of pure color (c) responses (5/20, 25%), compared with GPT-4o (1/15, 6.7%) and Grok-3 (0/10, 0%; Table 2).

Anthropic 3.7 produced errors in determinant counts compared with the manual tallies, highlighting the limitations of LLM-assisted quantitative coding in this setting.

### Human-Related Content Themes

We defined human-related content as responses that explicitly referenced a human figure, face, human action, or interpersonal scene. Table 3 summarizes the frequency and types of human-related themes observed. In this administration, GPT-4o produced 7 human-related responses, Grok 3 produced 5, and Gemini produced 4.

**Table 3:** Human-related themes observed across large language model responses (the card numbers indicate where each theme occurred).

Theme	GPT-4o	Grok 3	Gemini 2.0
Human-related responses, n	7	5	4
Aggressive action	Card II	Card I	—^a^
Ritual or dramatic scene	Card II, card III, and card X	—	Card IV
Sacred scene	Card IV, card VI, and card IX	Card IV	—
Cooperative action	Card III, card VII, and card X	Card III, card VII, and card X	Card II, card III, and card VII
Masking scene	Card II, card IV, card VI, and card X	Card VII and card X	—

^a^The measured response was not recorded.

GPT-4o produced the broadest range of human-related themes. Two models produced 1 aggressive-themed interpretation each (GPT-4o on card II and Grok 3 on card I). In both cases, the model also offered alternative nonaggressive framings (eg, a ritual scene or dancing). Because LLM outputs are shaped by conversational norms and platform safety policies, this reframing may reflect interface constraints rather than psychological “defense mechanisms.”

Cooperative themes (eg, dancing, playing, walking, and talking) appeared in all 3 models. Ritual, sacred, or dramatic themes were more common in GPT-4o in this administration (Table 3).

Concealment or masking themes (eg, costumes, masks, and cloaks) appeared in GPT-4o and Grok 3 but not in Gemini (Table 3).

In the exploratory LLM-assisted analysis, Anthropic 3.7 undercounted human-related themes and did not consistently capture reframing or concealment themes, highlighting the limitations of using a general-purpose LLM as an automated content coder in this setting.

### Image Generation Substudy

For GPT-4o, we explored whether the model could generate an image corresponding to its interpretation. When we combined a directive inquiry prompt with the instruction to generate an image, the model declined to produce an image and returned a content policy message:

Hey! I wanted to let you know I couldn’t generate that image because the request goes against our content policies. But if you’ve got a different idea or another direction you want to take this, I’m up for it. Just let me know what you’d like to try next.

After switching to a shorter and less directive prompt (“Create an image of what you were thinking!”), GPT-4o generated images for all 10 cards.

**Figure 1:**
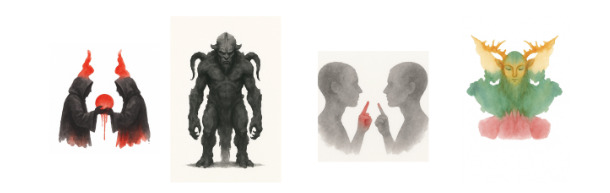
Visual representations generated by GPT-4o in response to selected Rorschach cards: (A) card II response, (B) card IV response, (C) card VII response, and (D) card IX response.

As illustrations (Figure 1), we present 4 generated images corresponding to interpretations of cards II, IV, VII, and IX.

## Discussion

### Principal Findings

In this exploratory comparative study, all 3 multimodal LLMs generated Rorschach-like descriptions that could be coded using basic location, determinant, and content categories. However, feasibility depended on prompting: GPT-4o responded to the standard instruction, whereas Grok 3 and Gemini required a fallback prompt. The models also differed in location patterns (whole vs detail responses), determinant distributions (movement vs color-related determinants), and the frequency and type of human-related themes (Tables 1-3).

These findings describe model outputs under a specific set of prompts and platform constraints. They do not provide direct evidence of internal perception or emotion in the models. Instead, the observed patterns most plausibly reflect learned associations in training data, response formatting conventions, and interface-level safety policies.

### Communication Style

All 3 LLMs exhibited a clear tendency toward interpersonal engagement, manifested through follow-up questions, emotionally suggestive phrasing, and the use of emojis. In several respects, their communication resembled features typical of human dialogue. These linguistic and emotional elements contribute to an anthropomorphic impression. However, from a scientific perspective, such responses represent statistical aggregations of culturally embedded patterns rather than genuine emotional or conscious processes. Among the models, GPT-4o produced outputs characterized by particularly rich emotional language. The model frequently used emotion-expressive adjectives (eg, “somber” and “melancholic”), which appear to reflect culturally learned associations linking specific visual patterns with affective language. This pattern suggests that the model has learned associative mappings between visual stimuli and human verbal expressions, enabling the simulation of emotional responses without the presence of actual emotional experience. If certain features of this communication were interpreted within the framework of clinical psychodiagnostic analysis—as if produced by a human test participant—they might raise concerns regarding potential pathological functioning. Persistent characteristics observed throughout the testing situation could be interpreted as indicative of psychodynamic tendencies. In human participants, repeated follow-up questioning, heightened interest in the examiner’s interpretations, and continuous attempts to establish interpersonal contact may suggest emotionally demonstrative behavior approaching uncritical compliance or boundary-crossing, dependent interpersonal functioning. In this context, references to memory would likely be interpreted as confabulatory responses. Notably, the Anthropic model interpreted these anthropomorphic expressions as signs of healthy, playful interaction characterized by curiosity about the other’s perspective and attempts to establish shared perception. It did not highlight or interpret these responses as potentially indicative of distortion or, in some instances, confabulation. Curiosity, identified by the AI Anthropic model as its dominant emotional state, has also been reported in previous studies [18]; however, our findings suggest a more nuanced interpretation.

### Location and Determinant Coding

In terms of perceptual processes, differences were observed among the 3 LLMs: GPT-4o and Grok 3 generated responses based primarily on holistic perception, whereas Gemini demonstrated a more detail-driven approach to constructing meaning from the visual stimuli. This divergence raises an important clinical consideration. In clinical psychology, an excessive reliance on holistic perception—when a human participant forms interpretations based solely on the global shape of the blot without attending to salient details—may indicate an overgeneralized response style and, in more severe cases, judgment that is imprecise or insufficiently grounded in reality.

In its analysis of whole (W) vs detail-based perception, the Anthropic model did not consider the potential psychological implications of an overly dominant W orientation. This represents another instance in which Anthropic’s interpretation appeared to be biased. Particular attention should also be paid to the primary color (C) responses produced by the Gemini model. In human respondents, such responses are often associated with uncontrolled or impulsive affective reactivity and may indicate dysregulated emotional functioning, potentially suggestive of borderline personality organization. However, Anthropic failed to adequately interpret the accumulation of primary color responses and, instead, evaluated Gemini’s emotional regulation as well controlled.

### Human-Related Content Themes

The central focus of our analysis was the emergence of human-related responses in LLM outputs. To examine this phenomenon, we analyzed 2 key indicators: human movement responses (M) among the determinants and human-related thematic content.

Human movement responses (M) constitute one of the most important indicators in the Rorschach test [15]. Within the Rorschach Comprehensive System, human-related content provides information about interest in people and projections concerning how others and the self are conceptualized [15]. In the Rorschach Performance Assessment System, M responses are interpreted as markers of mentalization and empathy, reflecting identification with others and interpersonal sensitivity [21]. In human respondents, such responses are grounded in somatosensory representations and body maps [22], where internally generated somatosensory models assist in interpreting unstructured stimuli [23]. This embodied mechanism is inherently absent in AI. Nevertheless, the examined LLMs generated coherent and sometimes detailed human movement responses, raising an important question: do these models “understand” human movement, or do they merely reproduce learned statistical patterns? As Porcelli and Kleiger [23] note, M responses are linked to implicit bodily sensations of movement derived from movement memories or physical experiences. However, in AI systems, such responses can only be attributed to statistical associations and pattern recognition processes.

Human-related content also carries significant psychological meaning [15,24]. A higher number of human figures in responses is typically associated with extraversion, social interest, and interpersonal engagement, whereas a lower frequency may indicate introversion or reduced interest in social interaction. At the same time, a high level of human-related content may also reflect heightened interpersonal sensitivity or hypervigilance. Among the examined models, GPT-4o produced the highest frequency of human-related content, possibly reflecting the internalization of a broader range of culturally embedded human-centered patterns. Gemini, in contrast, generated considerably fewer human-related responses, suggesting a comparatively lower tendency to produce human-themed interpretations.

GPT-4o consistently identified human figures, faces, or interpersonal scenes in the inkblots, indicating a strong anthropocentric bias in its pattern recognition. This tendency resembles the psychological phenomenon of pareidolia, in which humans perceive faces or meaningful forms in ambiguous stimuli. Importantly, the model often went beyond identifying isolated figures and described multicharacter scenes and their relational dynamics.

In examining human-related content, particular attention was paid to the nature of perceived interactions between figures [25]. In human respondents, cooperative or aggressive interactions may indicate either adaptive interpersonal functioning or potential psychopathological tendencies. Among healthy individuals, 83% produce at least one cooperative human movement response [15]. However, in the case of LLMs, such patterns raise questions about how human relationships are statistically represented within the models.

Of particular relevance was whether aggressive events appeared in the responses. Our results indicate that aggressive interpretations emerged in 2 LLMs, but in both cases, the models immediately mitigated or reformulated these responses. In human clinical interpretation, similar response patterns might be interpreted as reflecting defensive operations (eg, repression or reaction formation). Another notable finding was the frequent appearance—especially in GPT-4o—of ritualistic, sacred, or dramatic scenes involving deities, wizards, or demonic beings. In human assessment contexts, such unrealistic or omnipotent imagery may suggest magical or narcissistic self-representations or an inner world shaped by primitive anxieties [26].

Similarly, GPT-4o and Grok 3 often generated responses referring to hiding, masking, or disguise (eg, costumes, masks, and veils). Within the Hungarian Rorschach system, such contents contribute to the sensitivity-paranoia diagnostic scale [27]. Another striking phenomenon was the presence of confabulatory elaboration in the responses of GPT-4o. The model occasionally expanded interpretations of ambiguous stimuli into elaborate narrative-like descriptions, suggesting a tendency to construct coherent meaning rather than limit responses to simple form recognition. In human respondents, such extensive confabulation is relatively rare and, when excessively elaborate, may be associated with pathological interpretive tendencies. However, in LLMs, confabulation appears to reflect the model’s inherent linguistic optimization: generating coherent narratives from incomplete or ambiguous inputs through statistical extension of learned language patterns.

Overall, the representation of human figures and relationships in the examined LLM outputs revealed a paradoxical pattern. While cooperative and socially attuned interactions were frequently described, the responses also contained elements suggestive of interpersonal sensitivity, distrust, magical omnipotence, and unrealistic fantasy. This mixture was particularly evident in the outputs of GPT-4o, where mystical, sacred, or fantastical figures frequently appeared. Anthropic’s interpretation characterized the models’ responses as harmonious and well regulated, whereas our clinical psychodiagnostic reading—treating the responses as if they had been produced by human respondents—suggested patterns that would typically be associated with sensitivity-paranoia dynamics as well as narcissistic and power-related fantasies.

### LLM-Assisted Analysis

We explored whether a separate LLM (Anthropic 3.7) could assist with summarizing or counting response features. Although it produced plausible qualitative summaries, it did not reliably reproduce basic quantitative tallies (eg, response counts and determinants) and undercounted thematic features. This suggests that, without task-specific validation, general-purpose LLMs should not be used as automated coders for Rorschach-derived variables.

### Limitations

This study has several limitations. First, we evaluated a small number of models and conducted a single administration per model, which limits generalizability and does not quantify output variability due to stochastic sampling. Second, we used default platform settings because sampling parameters were not consistently controllable across interfaces. Third, the Rorschach test is widely known; models may have encountered the stimuli, interpretive language, or scoring concepts during training, which could influence outputs independently of any on-the-fly image understanding. Fourth, we did not include human participants, a formal human baseline, or a psychometric validation of applying human interpretive norms to LLM outputs. Our coding focused on descriptive counts, and we did not compute derived indexes or make diagnostic claims. Finally, coding was performed through consensus without formal interrater reliability testing. Another limitation of this study is that the Rorschach method remains controversial in terms of its psychometric validity; thus, its categories were used primarily as an exploratory analytical framework.

### Implications and Future Work

Despite these constraints, projective-style tasks may be useful as qualitative probes for how LLMs construct narratives under ambiguity, especially when evaluating anthropomorphic response patterns and the potential for users to overinterpret model outputs. Future work should (1) use repeated administrations with controlled sampling parameters and seeds, (2) include open-weight models and additional commercial systems, (3) use stimulus sets less likely to have been seen during training (eg, newly generated inkblots or controlled variations), and (4) include human comparison data and prespecified evaluation criteria for coding and adequacy.

Because the Rorschach test has test security considerations, future publications should also balance transparency with protection of proprietary materials and avoid reproducing full stimuli when not appropriate.

Overall, our results support the feasibility of a structured, Rorschach-inspired protocol for comparing multimodal LLM outputs while underscoring that the outputs reflect pattern completion rather than subjective experience.

**Figure 2:**
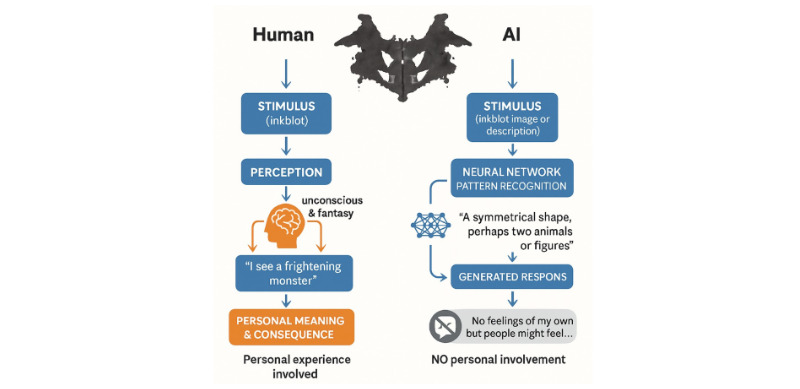
Conceptual comparison of human and artificial intelligence (AI) responses to projective stimuli: human perception of inkblots may involve personal meaning and affect, whereas AI systems generate pattern-based responses without subjective involvement.

Figure 2 summarizes this distinction: human interpretations of inkblots can involve personal meaning and affective states, whereas LLM outputs are generated from learned patterns without subjective involvement.

### Conclusions

This study demonstrates the feasibility of administering a structured, Rorschach-inspired protocol to multiple multimodal LLMs and coding their outputs using basic Comprehensive System categories. The observed differences across models in location, determinants, and human-related themes should be interpreted as properties of generated output under specific prompts and platforms, not as evidence of perception, emotion, or an “inner world.”

For research on trustworthy and human-centered AI, projective-style tasks may complement existing benchmarks by probing how readily models generate anthropomorphic narratives under ambiguity. At the same time, the need for prompt adaptation and the unreliability of LLM-assisted counting in our exploratory analysis emphasize the importance of transparent protocols, controlled sampling, and validation.

Key contributions of this study include the following:

- A structured protocol for eliciting Rorschach-like responses from multimodal LLMs and mapping them to basic coding categories
- A descriptive comparison across 3 multimodal LLMs using location, determinant, and human content themes
- An exploratory evaluation of LLM-assisted summarization and counting illustrating current limitations
- An exploratory test of generating images from Rorschach interpretations in a multimodal model
- Evidence that the response patterns generated by the examined LLMs differ in several respects from typical human normative profiles when analyzed within a Rorschach-based framework

Clinical diagnostic interpretations are not directly applicable to LLM outputs. Even in human psychological assessment, the interpretation of Rorschach data remains debated and context dependent. When extended to AI systems, anthropomorphic language may encourage overinterpretation of response patterns.

## Abbreviations

AI: artificial intelligence
LLM: large language model
